# Medical Criticism

**Published:** 1894-04

**Authors:** 


### Editorial.

### MEDICAL CRITICISM.

Upon another page of the present number we quote an editorial from the *New York Medical Record.* This is a very peculiar criticism of the dental profession, under the title of "The Defect in the Dentist." Ordinarily, the best answer to unjust charges is silence, permitting time to show that misstatements, however plausible, have only a limited life, and that eventually truth secures its rightful place.

Dentists have been so long accustomed to this kind of professional criticism that they have come to regard it very much as the chiding of a parent, a proper thing to give and to be received with all due humility.

It seems to us, however, that the time is past for quiet submission to this parental guidance and assumption of superiority.

Dentistry had its origin in the necessities of humanity. Medicine ignored it as part of its work. The world for uncounted centuries was left the victim of ignorance and to suffer untold agonies of misery, while this paragon of the healing art looked on and did nothing. It strenuously regarded the oral cavity as of no special importance in the treatment of disease. It seemingly held that the mouth and its appendages was simply an incident in the anatomy of the human body and unworthy the consideration of a dignified body of men such as the medical faculty of the world claimed for themselves. It practically refused to recognize that the teeth bore any direct relation to other organs of the body, or that more careful study of pathological conditions would be of any benefit to general medicine.

That there were honorable exceptions to this is admitted, yet the influence of John Hunter and the few who followed him with interest in the subject of odontology was so limited that it was not until the last half of the present century that medical practitioners began to appreciate the importance of attention to the intimate relations existing between the mouth and its connections, and it has only been in the last twenty years that they have deigned to treat the dentist with any evidence of respect or to accord him a position as one of the workers in the healing art.

This antagonizing influence forced the dentists of the earlier period to work apart from the medical fraternity and to build a distinct profession. It was a slow, laborious effort. The practical claimed first the attention of the laborers in this field. In this they had but little assistance from the older profession; on the contrary, they were regarded with thinly disguised contempt.

The time came when dentists organized their own schools of instruction, and slowly but steadily added branches of study until these schools began to assume a character that led medical practitioners to wonder, and from wondering to act. It became clearly evident to the belated understanding of this body that this young aspirant meant progress in every line of its work, and that sooner or later it would occupy a position worthy the respect of the world, whether it was recognized or not by professedly higher authority. It was further found that the schools established were becoming centres of attraction to young men who quickly recognized in their work a new source of profitable and intelligent employment.

The conservative medical element still regarded these evidences with disfavor, and persisted in believing these to be the vain efforts of a race of mechanics endeavoring to become semi-professional men. The younger element, though prejudiced by the elders, began to view this uprising in a different light, and while they reluctantly gave the right hand of fellowship, they could not disguise the fact that dentistry had made its place in science, and it was their duty as well as to their pecuniary interest to patronize it with limitations. The result has finally been that there is union everywhere with medical colleges and universities. Even conservative Europe has been obliged to make concessions to the spirit of progress and add to the great universities dental institutes. At present, therefore, there need be no question as to the position occupied by the modern dental operator.

It is to the credit of this young profession that it has never asked for recognition from the elder organization; in fact, it has rather resented any attempt to absorb it into the great body, feeling, very justly, that while there was much in common, medicine and dentistry must ever remain distinct in their organizations, and largely so in their work.

While this is true, it does not mean antagonism, but, on the contrary, entire harmony where the two professions meet on common ground, but entire independence in all other relations.

It is very late in the nineteenth century for an editor of a medical journal to feel it his duty to issue the screed quoted and to say, as he does in that editorial, that "The dentists of America are the best in the world, but they are, nevertheless, away behind the mark. We appreciate their ingenuity, their skill, their drills and dams, their composite fillings, and artificial teeth. They make the best kind of golden crowns, but do not deserve to wear them. This is because they do *not sterilize themselves, or teach their patients how to prevent caries.* They are wonderful patchers-up of things half gone, but they do not show us how to prevent the going."

It is true that dentistry does not yet "deserve to wear the crown," but not for the reasons stated. It has grown rapidly in the brief period of perhaps one hundred and fifty years, and is nearer that high position, or, to state it better, is based more nearly on scientific lines, than medicine in its centuries of work. In that time it has quite thoroughly explored its limited field, but has never yet worn the collar of subserviency to authority which has held medicine in bonds to an extent that progress has been almost an impossibility until a comparatively recent period. Change came with additional light thrown upon the origin of disease through bacteriological investigations, but previous to this time, barely two decades, medicine practically lived on the teachings of the past, and wore its collar with amazing equanimity.

While medicine was asking, with bated breath, what changes the theory of the germ origin of disease was likely to produce, if perchance it should be proved true, dentists of intelligence were adapting themselves to the new conditions, and before medical men had come to realize the importance of these new ideas, had adopted them in practice and had demonstrated that their only hope of success in the treatment of pathological conditions was through antisepsis. Yet our critic boldly charges that "they do not sterilize themselves," and, by inference, would lead us to believe that medical men universally are careful in this respect. That there is too much carelessness in both departments is without doubt true, but it is not true of the better elements of either medicine or dentistry.

The truth is, that the importance of sterilization is not as yet fully appreciated as it should be. It is no unusual thing to find the medical attendant making no attempt to sterilize his hands in his daily rounds, and we have seen the temperature taken by the mouth, the thermometer simply rinsed in water, and replaced in its case, to be used on some other patient. That this is common we do not believe, but it exists, and illustrates the fact that dental carelessness is not exceptional.

Further our critic says, "There is hardly a dentist (there are a few) who works in an aseptic way or uses aseptic instruments." The great importance of rendering instruments aseptic is as thoroughly understood in dentistry as it is supposed to be in medicine, and no dentist of any character would dare to practise without using every precaution available. There are many difficulties in the way of doing this thoroughly, and these have not been entirely surmounted. The instruments can be rendered harmless, but the hands cannot be treated in the same way, or be considered aseptic in the best meaning of that term. We have great faith in thorough cleanliness, which can be accomplished by a free use of soap and water.

Our critic forgets, perhaps, that some of the best surgeons regard cleanliness as the true antiseptic, for they argue that a germicide sufficiently powerful to destroy pathogenic germs will destroy equally the tissue on which it is placed. Hence all that can be hoped for, as far as dentists hands are concerned, is to render them incapable of harm, and this can be accomplished by inhibiting the development of pathogenic bacteria.

Our critic lays great stress on the fact that dentists do not show patients "how to prevent caries." Is he aware that this disease of the teeth is the result of fermentation through microbic action? Is he further aware that caries is dependent on many factors which no amount of care or instruction to patients will prevent? It is not probable that he knows anything about it, and hence ignorantly assumes that the dentist can accomplish an impossibility. The most that can be done is to minimize destruction. His idea of what is needed to accomplish this desirable result is good as far as it goes, and it is true that "an antiseptic wash is needed" in every case; but will this make chalky teeth dense, or hinder the impaction of food on impinging surfaces, or prevent patients from eating all sorts of injurious foods, or prevent the medical attendant from ordering acid remedies without any attention to neutralization, or will it indicate to the same learned caretaker of the health of the people that sanitary attention to the mouth is an important consideration in cases of long-continued fever. If these and more are mastered, he will, possibly, come to realize that caries cannot be prevented absolutely, and that the prophylactic treatment to this end has never yet been devised and, probably, never will be.

In return for his kindly suggestion, we would ask him to remember, using his own language, "that the mouth is a rich culture-ground for microbes," and that to secure, in a measure, immunity from many diseases it would be well to pay some attention to inhibiting the development of pathogenic bacteria there and in the nasal tract, and thus prevent them from reaching vital organs. It has long been our opinion that medical men are grossly negligent of duty in this respect, especially where contagion is to be feared. They will talk learnedly of sterilization of hands and instruments, yet never a word in regard to "this rich culture-ground," the poison manufactory, so to speak, for the entire system. They will sterilize the excreta with a persistence worthy of honorable mention, but they allow the development of death-dealing germs in the mouth to proceed unheeded. Why, may we ask, has medicine so long neglected this opportunity to meet disease at its inception?

The period of criticism of this kind has passed. Dentistry has come as one of the great needs of humanity. It has been developed beyond the stage of mechanism into the region of scientific acquirement, in no degree inferior to that possessed by others. Whether it will be absorbed in medicine, as some think, or remain an independent profession, as we view it, it holds, and will continue to maintain, a position equal to that to which our critic belongs, in thoroughness of manipulation, in effectiveness of treatment, and in excellence in results within its range of work.

Its management of the oral cavity in every direction has increased the tenure of human life and has almost obliterated the feebleness of three score years and ten. It has promoted and continued the digestive functions unimpaired far beyond former periods, and by maintaining a more perfect balance of health has preserved mental vigor. In a word, old age has almost ceased, through its intelligent skill, to have the terrors formerly anticipated. The truth of this record cannot be controverted, and upon this we rest as sufficient for our honor, and in the development of which medicine has had no part.

